# Super-enhancer-associated INSM2 regulates lipid metabolism by modulating mTOR signaling pathway in neuroblastoma

**DOI:** 10.1186/s13578-022-00895-3

**Published:** 2022-09-16

**Authors:** Haibo Cao, Ran Zhuo, Zimu Zhang, Jianwei Wang, Yanfang Tao, Randong Yang, Xinyi Guo, Yanling Chen, Siqi Jia, Ye Yao, Pengcheng Yang, Juanjuan Yu, Wanyan Jiao, Xiaolu Li, Fang Fang, Yi Xie, Gen Li, Di Wu, Hairong Wang, Chenxi Feng, Yunyun Xu, Zhiheng Li, Jian Pan, Jian Wang

**Affiliations:** 1 Institute of Pediatric Research, Children’s Hospital of Soochow University, No. 92 Zhongnan Street, Suzhou, 215003 China; 2 Department of Pediatric Surgery, The Affiliated Hospital of Yangzhou University, 225000 Yangzhou, Jiangsu China; 3 Department of Pediatric Surgery, Children’s Hospital of Soochow University, No. 92 Zhongnan Street, Suzhou, Jiangsu 215025 China; 4 Medical College of Soochow University, Suzhou, Jiangsu 215123 China

**Keywords:** Super-enhancer, INSM2, Lipid metabolism, Neuroblastoma, mTOR, SREBP1

## Abstract

**Background:**

Abnormal lipid metabolism is one of the most prominent metabolic changes in cancer. Studies have shown that lipid metabolism also plays an important role in neuroblastoma. We recently discovered that the insulinoma-associated 2 gene (INSM2) could regulate lipid metabolism in neuroblastoma (NB) and is improperly controlled by super enhancers, a mammalian genome region that has been shown to control the expression of NB cell identity genes. However, the specific molecular pathways by which INSM2 leads to NB disease development are unknown.

**Results:**

We identified INSM2 as a gene regulated by super enhancers in NB. In addition, INSM2 expression levels were significantly upregulated in NB and correlated with poor prognosis in patients. We found that INSM2 drives the growth of NB cell lines both in vitro and in vivo. Knocking down INSM2 inhibited fatty acid metabolism in NB cells. Mechanistically, INSM2 regulates the expression of SREBP1 by regulating the mTOR signaling pathway, which in turn affects lipid metabolism, thereby mediating the occurrence and development of neuroblastoma.

**Conclusion:**

INSM2 as a super-enhancer-associated gene could regulates lipid metabolism by modulating mTOR signaling pathway in neuroblastoma.

**Supplementary Information:**

The online version contains supplementary material available at 10.1186/s13578-022-00895-3.

## Background

Neuroblastoma(NB) is a frequent solid tumor in children. Its clinical manifestations are diverse. This characteristic depends on the biological characteristics of the tumor to a great extent [1]. Studies have shown that NB originates from the precursor cells of sympathetic adrenal system derived from neural crest, which is easy to occur in boys, and is highly related to ethnic differences. The genetic and epigenetic basis of this phenomenon is still unclear [2]. The clinical manifestations of NB are specific. NB frequently regresses spontaneously in patients 18 months of age, and with increasing age, it manifests as rapid progression or even death [3]. With advances in gene sequencing technologies and improved bioinformatics tools, it has gradually emerged that heterogeneity in NB heterogeneity can be explained by some repeated molecular abnormalities. These genomic alterations include MYCN amplification, ALK mutations, and chromosome copy number aberrations [4]. Variants in these genomes maintain the biology of the NB.

Enhancers are segments of DNA sequence on the genome that affect cell fate by regulating the precise spatiotemporal expression of targeted genes during cell development and differentiation, and they are important cis-acting regulatory elements for multicellular organisms’ cell identity and development processes.In 2013, the Richard A. Young laboratory proposed the concept of super-enhancers (SEs) based on research on enhancers at the time [5]. The large cluster of transcriptionally active enhancers enriched with a high density of key transcription factors (Master transcription factors), cofactors (Cofactors), and enhancer apparent modification markers (Histone modification marks) can explain cell type-specific expression patterns. It has demonstrated significant promise in the pathogenesis of developmental biology and cancer [6, 7]. The identification of super-enhancers was based on differences in the strength of the binding levels of enhancer transcriptionally active marker molecules, including cofactors (e.g., Mediator and cohesin), histone modification markers (e.g., H3K27Ac and H3K4me1), chromatin-modifying molecules (e.g., p300), etc. [6, 7]. Therefore, Hnisz et al. further analyzed whether other molecular markers of enhancers could be applied to the identification of super-enhancers, showing that H3K27Ac is the best choice for the further identification of super-enhancers of 86 human cells and tissues, including diseases such as tumors[7]. In different tumors [6], including neuroblastoma (NB) [8–10], super-enhancers are frequently abundant at critical oncogenes. Recent research suggests that super-enhancer-associated genes such HAND2, MEIS2, GATA3, and PHOX2B play a growing role in NB cell identity [11–13]. In previous studies, we performed Chip-seq testing on neuroblastoma cell lines against histone H3K27Ac, screened a series of possible NB-associated super-enhancers and the genes most likely regulated by them, and studied them deeply.

The insulinoma-associated 2 gene (INSM2), also known as IA-6 or MLT1. Previous studies showed that it was closely associated with the differentiation of pancreatic endocrine cells [14]. But how it works in the human NB has not yet been reported.

Emerging evidence showed that the level of lipid synthesis was increased significantly in human cancer cells to meet the demand for the enhanced membrane biogenesis [15, 16]. The utilization and storage of lipids by malignant tumor cells is also elevated [17]. Targeted modulation of lipid metabolism pathways may have great clinical implications in anti-cancer therapy [18]. As a result, tackling the molecular basis of lipid metabolism in neuroblastoma cells could provide clues for targeted interventional therapy. In this study, we verified a new super enhancer-regulated gene INSM2 which is highly expressed in clinical NB specimens. INSM2 could affect lipid synthesis in NB cells by modulating mTOR signaling pathway, and affect cell survival and expansion by regulating some key genes such as MYCN [19, 20] and ANXA2 [21] in NB cells. The molecular foundation of INSM2 regulating fatty acid metabolism in NB was first demonstrated in our study.

## Results

### Identification of INSM2 as a super-enhancer-associated gene in NB

**Fig. 1:**
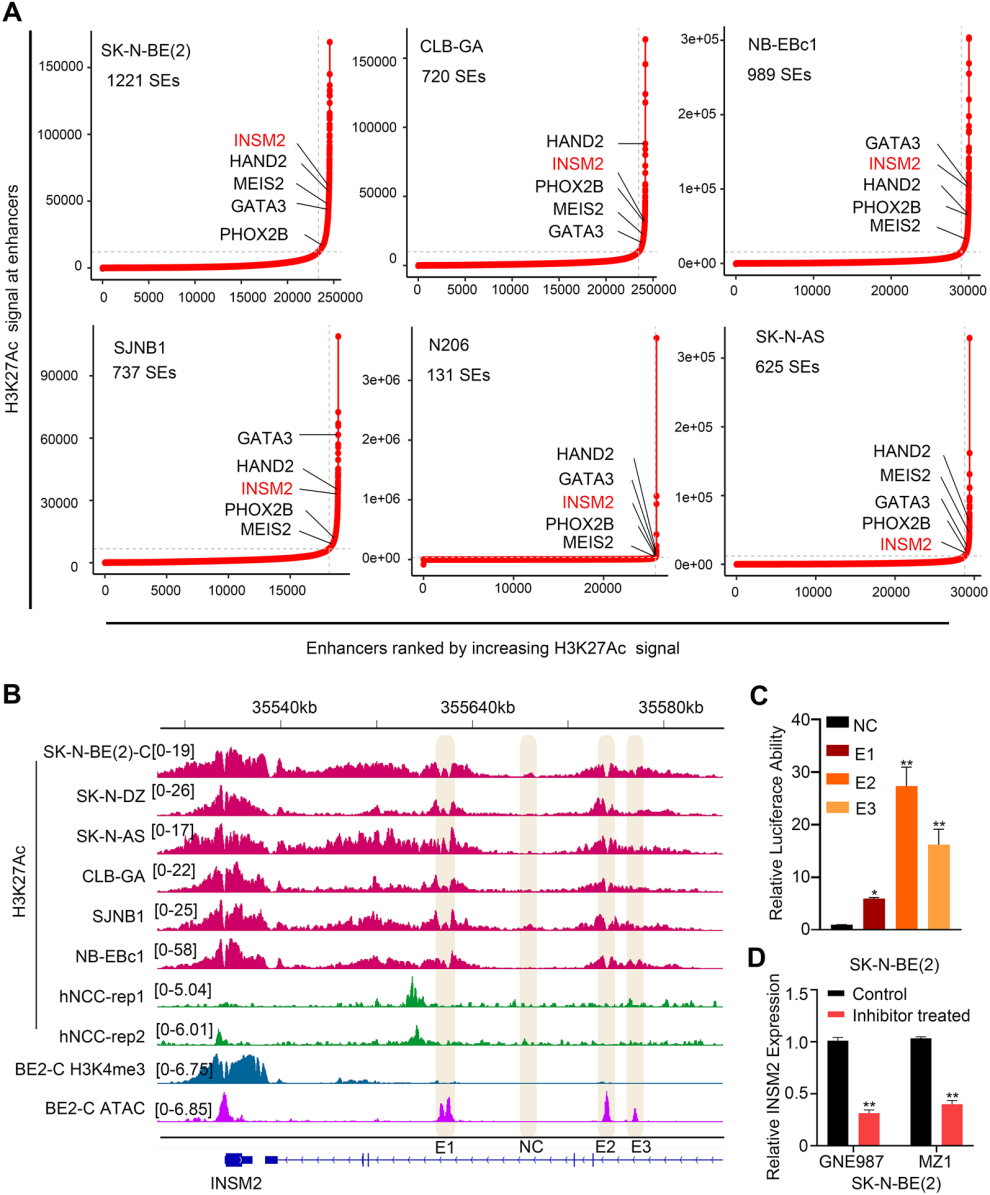
Identification of INSM2 as a super-enhancer-associated gene in NB.** A** Enhancers were ranked by increasing H3K27Ac signal in 6 NB cell lines. The hockey stick plots shown genes associated with super-enhancers. **B** The ChIP-seq gene tracks represent the H3K27Ac signal in NB cell lines, and neural crest cells, while the last two tracks represent H3K4me3 and ATAC signals in BE(2)-C at the INSM2 gene loci. The y-axis represents the total number of mapped reads per million, while the x-axis represents the linear sequence of genomic DNA. **C** Activity of the dual luciferase reporter vector containing NC or super- enhancer motifs that were transfected into SK-N-BE(2) cells. **D** q-PCR of INSM2 expression in SK-N-BE(2) cells treated with GNE987 and MZ1. GAPDH served as an internal reference. Unpaired two-sided t-test was used for the analysis in **C**. ns, not significant; *P < 0.05; **P < 0.01. The data are based on three separate experiments

First, we performed H3K27Ac analysis on ChIP-seq datasets from six neuroblastoma cell lines(SK-N-BE(2), CLB-GA, NB-EBc1, SJNB1, N206, SK-N-AS). We sorted all putative enhancers by increasing H3K27Ac signal and filtered out enhancers with substantial H3K27Ac enrichment using these data. The result showed that INSM2 and a number of known NB oncogenes such as PHOX2B, MEIS2, GATA3 and HAND2 [11] were enriched (Fig. 1A). Following that, we found three potential INSM2 enhancers (E1, E2 and E3) (Fig. 1B). These enhancer elements were then cloned into the pGL3-promoter luciferase reporter vector, which was then transfected into SK-N-BE(2) cells, along with a control region. The Luciferase reporter assay showed robust luciferase reporter activities of E2 and E3, but weak activity of E1 (Fig. 1C), consistent with the high H3K27Ac signal in E2 and E3, but not in E1. BRD4 is an epigenetic regulator known to have a role in cancer super-enhancer assembly and transcriptional control [22, 23]. GNE987 and MZ1 disrupted the binding of BRD4 to acetylated chromatin, thereby inhibiting cancer growth and inducing apoptosis. We therefore analyzed INSM2 expression following GNE987 and MZ1 treatment in NB cells. qPCR showed that the expression of INSM2 significantly decreased in SK-N-BE(2) cells treated with GNE987 and MZ1 (Fig. 1D).

### INSM2 is highly expressed in NB

**Fig. 2:**
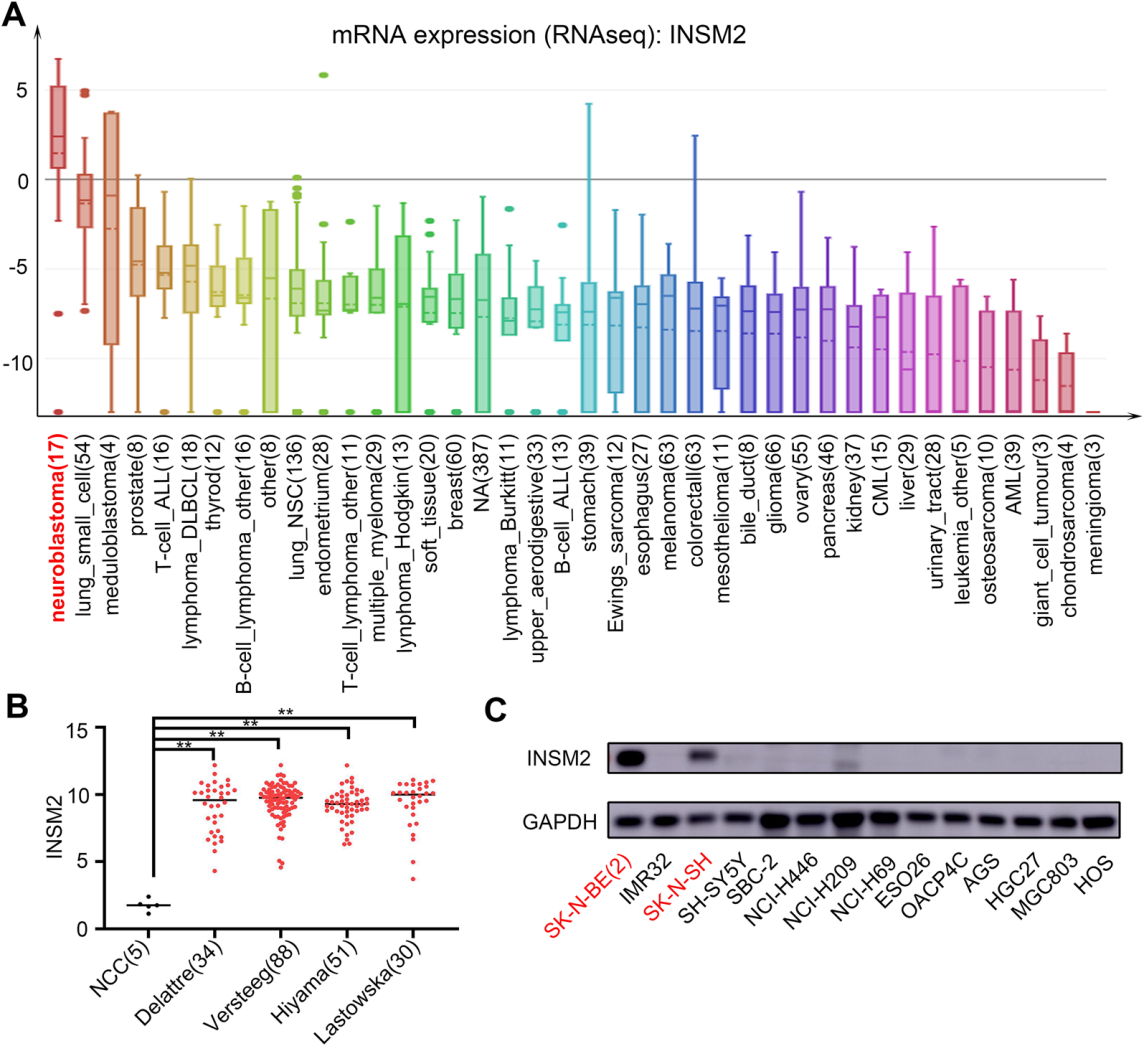
INSM2 is highly expressed in NB. **A** Data retrieved from the CCLE project depicting mRNA expression of INSM2 across various types of human cancer cells. **B** The expression of INSM2 in normal human trunk neural crest (NCC, GSE14340, *n* = 5) and four different neuroblastoma cohorts (GSE14880, *n* = 34; GSE16476, *n* = 88; GSE16237, *n* = 51; GSE13136, *n* = 30). y-Axis represents the normalized log2 expression value. Data are represented as mean ± SD. **P < 0.01. **C** Western blot indicating the expression of INSM2 in NB cell lines and in cell lines of other tumors

Based on our screening experiments, we predicted that the INSM2 gene may play an important role in NB. Therefore, we next investigated whether gene and protein expression levels of INSM2 are associated with NB in cellular and clinical samples. We retrieved INSM2 mRNA expression data from more than 40 tumor cell lines (data from CCLE) and found that NB had the highest INSM2 expression (Fig. 2A). Next, we compared the expression of INSM2 in human normal neural crest cells and four different neuroblastoma cohorts. We found that the expression of INSM2 in neuroblastoma was much higher than that in normal neural crest cells (Fig. 2B). For a more objective comparison, we found a recent study [24] which performed RNA-Seq on ganglioneuroma (GN) and NB samples. We analyzed the data (GSE147635) and found that INSM2 was also significantly highly expressed in NB in comparison to GN samples (Additional file 1: Fig. S1). We also used western blotting to quantify the protein expression of INSM2. As Western blot shows, INSM2 protein is specifically expressed in NB cell lines (SK-N-BE(2) and SK-N-SH) compared to multiple tumor cell lines (Fig. 2C). These results clearly show that INSM2 is elevated in NB and may behave as a potential oncogene. As a result, we investigated INSM2’s function in NB further.

### NB cell growth is inhibited after knockdown of INSM2 ***in vitro*** and ***in vivo***

**Fig. 3:**
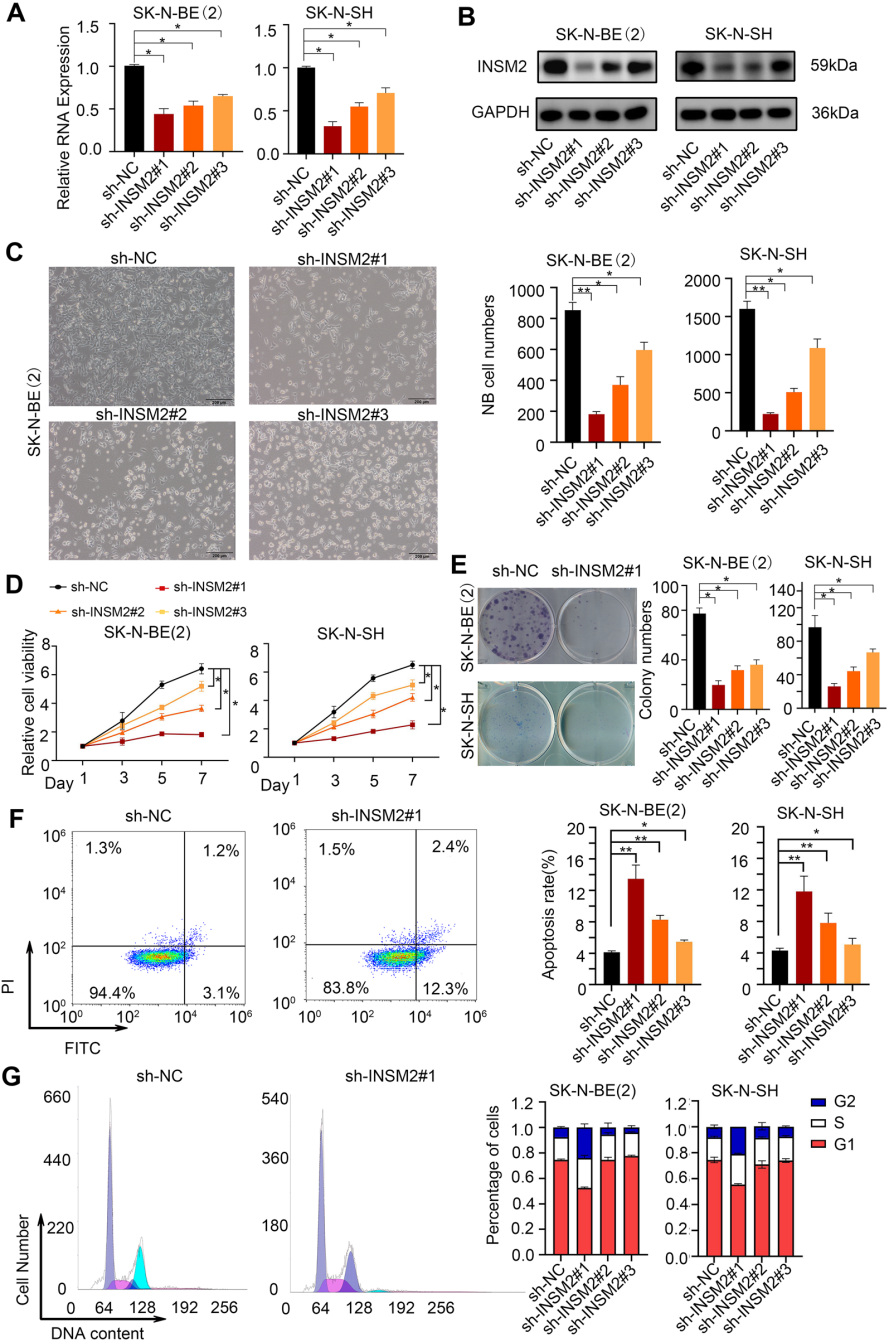
INSM2 promotes survival and expansion of NB cells ***in vitro***. **A, B** western blot analysis(**A**) and q-PCR (**B**) of INSM2 expression in SK-N-BE(2) and SK-N-SH cells infected with sh-NC or sh-INSM2s. GAPDH served as an internal reference. **C** Representative images of NB cells infected with sh-NC or sh-INSM2s. Scale bars: 200 μm. **D**-**G** SK-N-BE(2) and SK-N-SH cells were infected with sh-NC or sh- INSM2s. The cells were harvested for CCK-8 colorimetric assay (**D**), colony formation assay(**E**), cell apoptosis assay (**F**), periodic assay (**G**) after stable cancer cells were established. For **A, D–G**, representative images are shown in the left and quantification in the right. Each sh-INSM2 group was compared with the sh-NC group. One-way ANOVA with Bonferroni correction in **D**; unpaired two-sided t-test in **A, E–G**; ns, not significant; *P < 0.05, **P < 0.01. The data are based on three separate experiments

We investigated INSM2 function by knocking down its expression in NB cell lines and confirming knockdown efficacy using qPCR (Fig. 3A) and western blotting (Fig. 3B). As seen under the microscope, the knockdown of INSM2 resulted in a significant inhibition of cell proliferation in two NB cell lines (Fig. 3C). In comparison to sh-NC transfection, INSM2 knockdown dramatically decreased NB cell proliferation in the CCK-8 assay (Fig. 3D) and colony formation assay (Fig. 3E). Furthermore, apoptotic experiments suggested increased SK-N-BE(2) apoptosis after INSM2 was knocked down (Fig. 3F), and cell cycle experiments also showed that cell division stagnated in phase G2 (Fig. 3G). In order to determine whether the phenotypes resulting from INSM2 knockdown can be reversed, we designed an INSM2 high expression plasmid and infected SK-N-BE (2) and SK-N-SH cells, respectively. Through the comparison of cell proliferation, we found that the proliferation rate of sh-INSM2#1 + INSM2 group was significantly faster than that of sh-INSM2#1 group (Additional file 1: Fig. S2). Therefore, we conclude that INSM2 may be positively regulated by neuroblastoma cell growth.

**Fig. 4:**
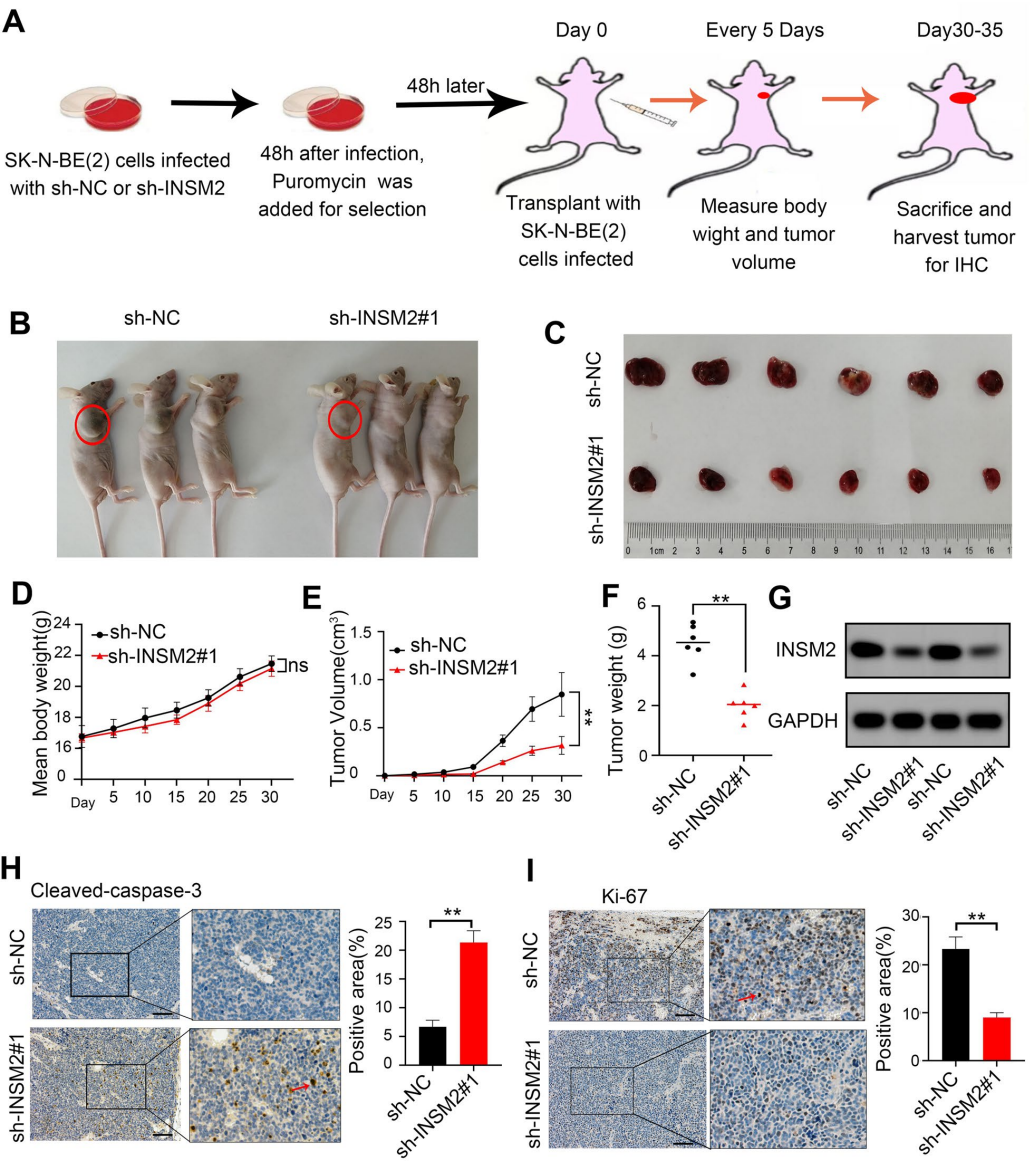
INSM2 promotes NB growth ***in vivo***. **A** Flowchart showing the experimental design used to obtain subcutaneous xenografts in nude mice; **B** Representative nude mice images of xenografts formed by subcutaneous injection of SK-N-BE(2) cells stably transfected with sh-NC or sh-INSM2#1 through the armpit of nude mice (*n* = 6). **C** Representative tumor images. **D** Weight growth curve of nude mice after injection. **E** Growth curves of tumor in vivo. **F** Tumor weight at the end points of xenografts(*n* = 6). **G** Western-blot of living tumor tissues. **H–I** IHC analysis for Cleaved Caspase-3 (**H**), Ki-67(**I**), expression was performed in sections of tumors harvested from xenografts, and percentages of the positive cells were quantified. All data are shown as mean ± SD (scale bars of **H**, **I** = 50 μm). The sh-NC group was compared to the sh-INSM2#1 group. One-way ANOVA with Bonferroni correction in **D, E**; unpaired two-sided t-test in **F, H–I**. NS, not significant; *P < 0.05; **P < 0.01. The data are based on three separate experiments

To further assess whether INSM2 knockdown could suppress tumor growth in vivo, we conducted a nude mouse xenograft model by subcutaneous injection of INSM2 knockdown NB cells (Fig. 4A). When compared to controls, INSM2 knockdown significantly slowed tumor growth and reduced tumor volume (Fig. 4B, C, E, F). Meanwhile, the treatment group and control group had no remarkable difference in mouse body weight (Fig. 4D). The knockdown efficacies in subcutaneous xenograft tumors were verified by Western Blot (Fig. 4G). IHC analysis showed that knockdown of INSM2 increased the apoptotic rate (Cleaved Caspase-3, Fig. 4H), reduced the proliferation (Ki-67, Fig. 4I) and reduced angiogenesis (CD31, Additional file 1: Fig. S3) of the xenografts.

### INSM2 represses hallmark cholesterol homeostasis and hallmark adipogenesis in NB cells

**Fig. 5:**
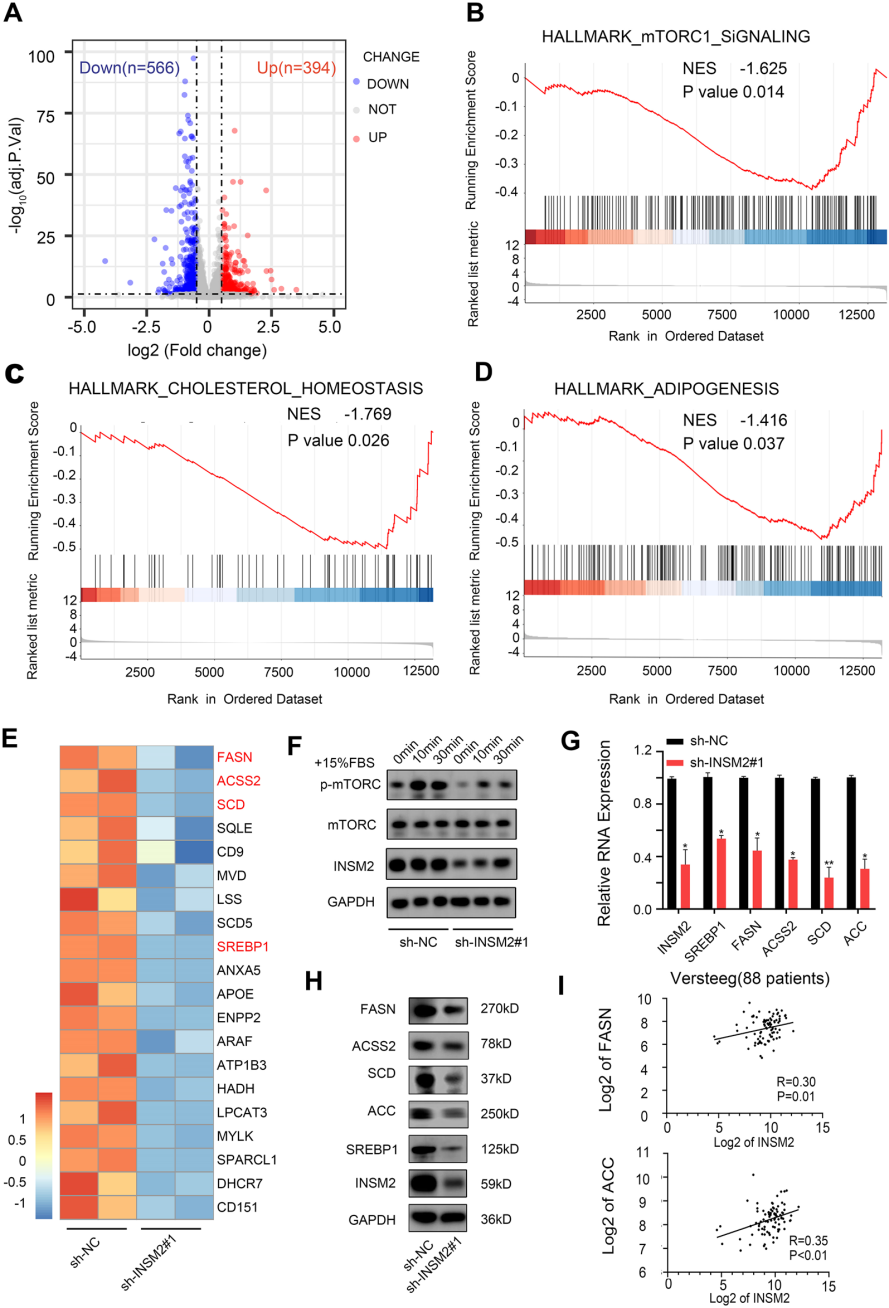
INSM2 is involved in the regulation of mTORC1, Cholesterol and Adipogenesis signaling pathway.** A** Volcano plot showing differentially expressed genes in INSM2-knockdown SK-N-BE(2) cells. Genes with abs(log2(foldchange)) > 0.5 and adjusted p-value < 0.05 were considered significant. **B–D** GSEA plots show alterations in mTORC1, CHOLESTEROL and ADIPOGENESIS signaling pathways in INSM2-knockdown SK-N- BE(2) cells. **E** Heatmap showing the expression of cancerrelated genes differentially expressed in INSM2-knockdown SK-N-BE(2) cells. **F** WB showing that mTORC phosphorylation is inhibited after INSM2 knockdown. **G** qPCR analysis of mRNA levels for the genes related to fatty-acid synthesis in SK-N-BE(2) cells transfected with sh-NC or sh- INSM2#1. **H** Fatty acid synthesis related protein levels decreased. **I** Scatter plot showed correlations between INSM2 and FASN, ACC mRNA level in tumors of 88 NB patients investigated. Unpaired two sided t-test for analysis in **G**. NS, not significant; *P < 0.05; **P < 0.01. The data are based on three separate experiments

To investigate potential downstream genes of INSM2 in neuroblastoma, we performed RNA-seq analysis on INSM2 knockdown and control SK-N-BE(2) cells. In INSM2 knockdown SK-N-BE(2) cells, 566 genes were downregulated, and 394 were upregulated in INSM2 knockdown SK-N-BE(2) cells (log2 Fold Change ≥ 0.5, *p* < 0.05) (Fig. 5A; Additional file 2). GSEA showed that genes producing changes were enriched for repression of mTOR signaling, hallmark cholesterol homeostasis and hallmark lipogenesis (Fig. 5B–D). The differential expression of the specific cancer-related genes driving the enrichment results was further elucidated by heatmap analysis (Fig. 5E). Next, we confirmed that knockdown of INSM2 inhibited the phosphorylation of mTOR by Western blot (Fig. 5F). Then, through qPCR (Fig. 5G) and Western blot (Fig. 5H), we confirmed alterations in expression of selected genes that have been shown to be involved in lipid metabolism, such as SREBP1, FASN, ACC, ACSS2, and SCD. Studies have shown that neuroblastoma cell viability has decreased significantly after interfering with fatty acid anabolism [25], suggesting that lipid metabolism also plays an important role in neuroblastoma.

Next, we also performed further experiments to demonstrate whether lipid metabolism affects the growth of NB. We first investigated the effect of FASN-IN-4 (HY-12,648, Medchemexpress), a potent inhibitor of FASN, on NB growth. After determining its IC50, we selected the appropriate concentration in vitro based on its ability to degrade FASN protein. The results showed that after inhibiting the lipid synthesis of NB cells, the cell viability was significantly decreased. Then we constructed the SK-N-BE(2) cell line with stable knockdown of FASN. The results also showed that after inhibiting the lipid synthesis of NB cells, their cell viability was significantly decreased. The main transcription factor regulating cholesterol and fatty acid biosynthesis is SREBP1 (Sterol regulatory element binding protein 1) [26]. And recently, studies have also shown that SREBPs are regulated by mTOR signaling [27].

In addition, KEGG analysis revealed that these genes were significantly enriched for several cancer-associated pathways (Additional file 1: Fig. S5A, Additional file 3). After knocking down the expression of INSM2 in NB cells, some important oncogenes of NB, such as MYCN and ANXA2, were significantly decreased, which we also verified by qPCR and Western blot (Additional file 1: Fig. S5B, C). Collectively, the above findings suggest that INSM2 may promote NB progression through by targeting mTOR signaling to regulate genes involved in lipid ab initio anabolism and regulating other neuroblastoma oncogene.

### Clinical positive correlation between INSM2 and lipid metabolism-related genes

Through analysis of the Versteeg database, we found a positive correlation between INSM2 and lipid metabolism genes (FASN and ACC) in 88 NB patients (Fig. 5I; Additional file 4). To explore clinical relevance, we evaluated the association of fatty acid synthesis-related genes with overall survival in NB patients in three independent datasets [Versteeg (88 patients), Kocak (476 patients) and NRC (283 patients)]. All three datasets suggested that high expression of FASN, ACC, ACSS2 and SCD was associated with reduced overall survival (Additional file 1: Fig. S6A–D).

### Knocked down of INSM2 inhibited fatty acid metabolism in NB cells

**Fig. 6:**
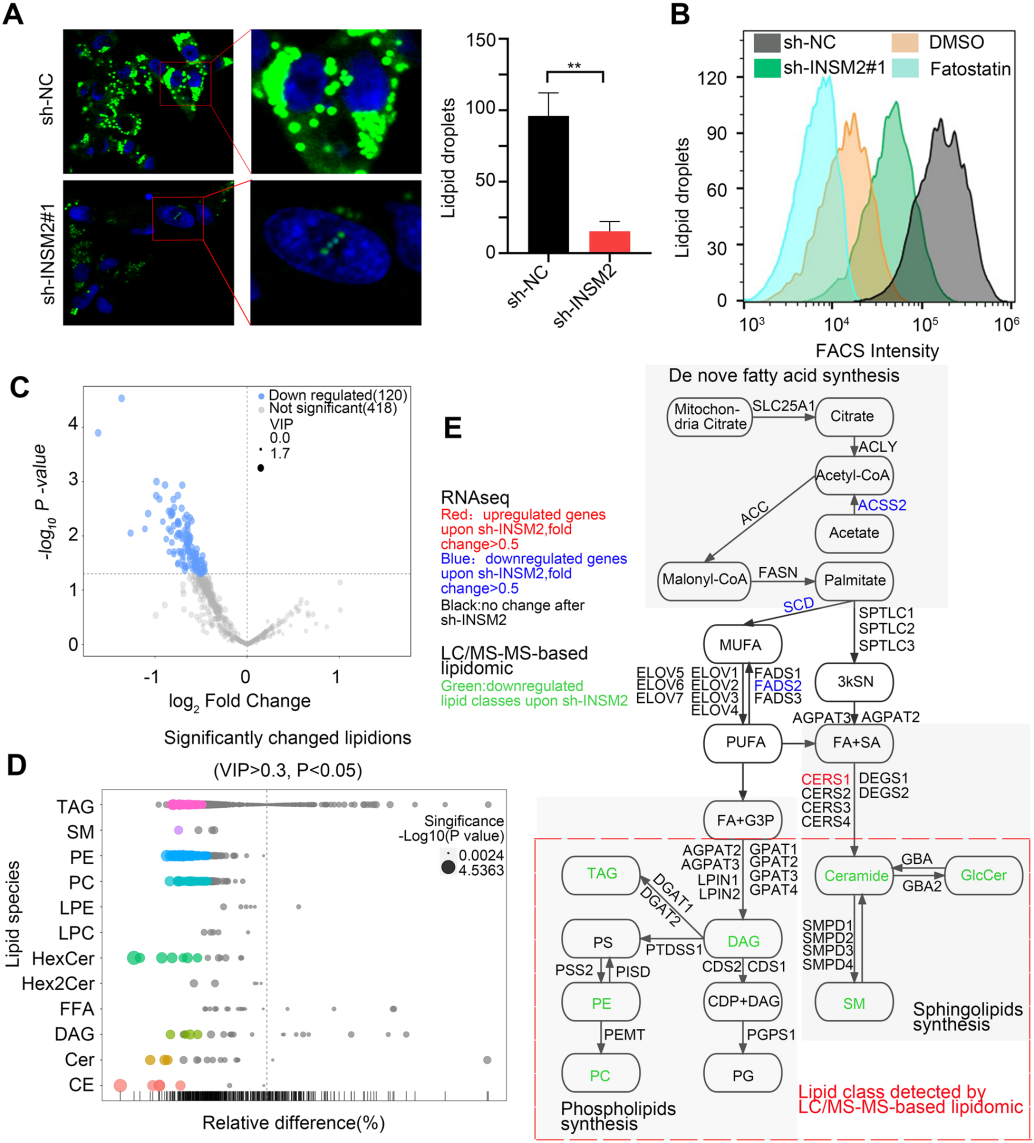
INSM2 regulates fatty acid synthesis in NB cells. **A** Confocal images(left) of lipid droplet and quantification(right) in INSM2-knockdown SK-N-BE(2) cells. **B** Flow cytometry analyses of lipid droplet in SK-N-BE(2) cells stably transfected with sh-NC or sh-INSM2#1.DMSO and fatostatin treated SK-N-BE(2) cells were used as positive controls. **C** Volcano plot of LC/MS-MS–based lipidomics after INSM2 knockdown. Each dot represents a lipid species. **D** The lipid group Bubble Diagram showed the changes of lipid metabolites in INSM2-knockdown SK-N-BE(2) cells. Each dot represents a metabolite. **E** Schematic diagram showing the regulation of lipid synthesis pathways by INSM2 via integration of RNA-seq, and lipidomics data. Unpaired two-sided t-test was used for the analysis in a. ns, not significant;*P < 0.05; **P < 0.01. The data are based on three separate experiments

**Fig. 7:**
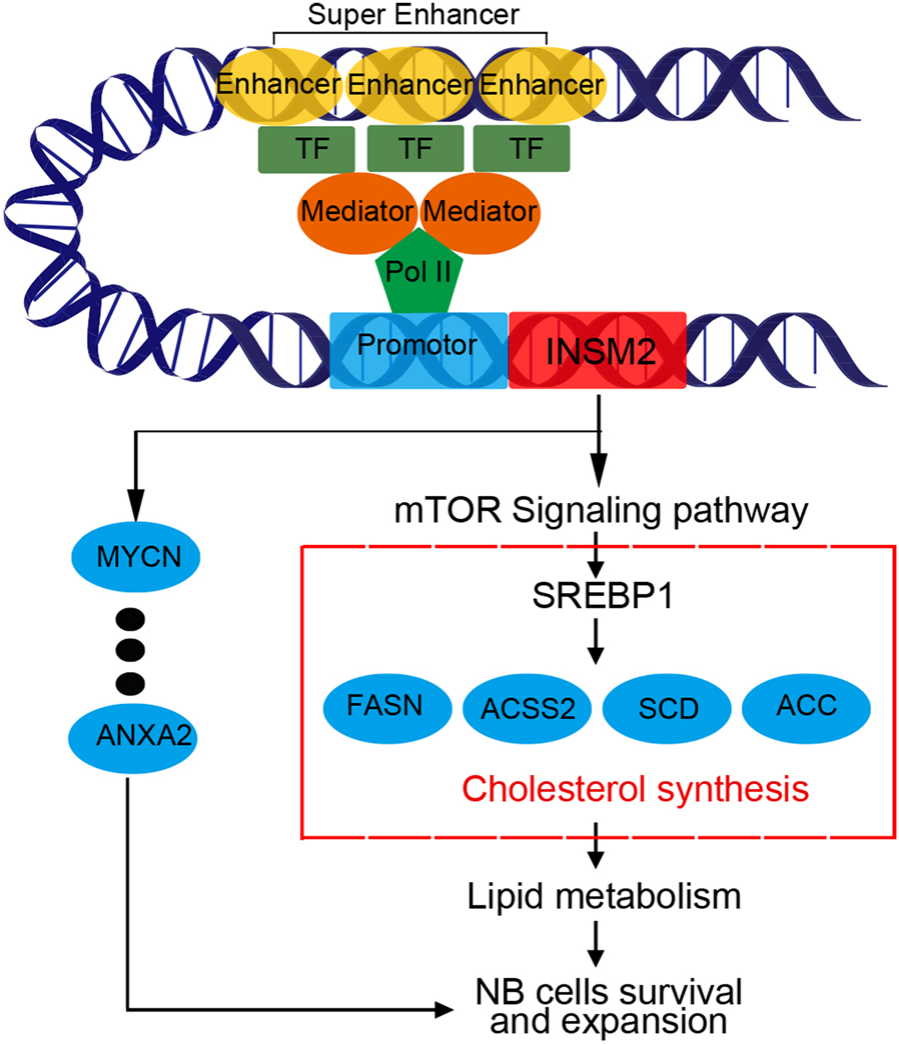
Proposed model of the mechanism by which super-enhancer-associated INSM2 promotes neuroblastoma progression

We further investigated how INSM2 plays a role in fatty acid metabolism. Explicit decrease in intracellular lipid droplet content after knockdown of INSM2 in neuroblastoma. The changes in lipid droplet content in NB cells after INSM2 knockdown were analyzed by immunofluorescence assay (Fig. 6A) and flow cytometry (Fig. 6B). Fatostatin, a fat metabolism inhibitor, and DMSO-treated NB cells were used as positive controls. The results all showed an explicit decrease in intracellular lipid droplet content after knockdown of INSM2 in NB cells. We then performed full quantitative lipomic analysis on human neuroblastoma cells to determine the types of lipids that change in NB cells after knocking down INSM2. We performed robustness analysis of control and INSM2 knockdown cell samples (*n* = 6) and high correlation between replicates was achieved (Pearson correlation coefficient ranging from 0.84 to 0.86; the lipidomics approach has high sensitivity and lipidome coverage) [28]. We quantified a total of 538 lipid ions belonging to 12 lipid classes (Additional file 5). This quantification showed that INSM2 knockdown resulted in a significant reduction of 120 lipid ions in NB cells (Fig. 6C). We found that many lipids involved in the metabolism of lecithin (such as DAG, TAG, CDP-DAG, PE, and PC) and sphingolipids (such as SM and GlcCer) were reduced after INSM2 depletion (Fig. 6D). In addition, integrative analyses of RNA-seq and lipidomics data identified INSM2 downstream factors and associated lipid (Fig. 6E). Some of these down-regulated lipids (sphingomyelin and phospatidylcholine) have been found to have pro-survival or anti-apoptotic functions in certain cancer cells [29]. Therefore, INSM2 may regulate the growth and survival of NB cells by regulating the synthesis of the above-mentioned lipids. In conclusion, we speculate a mechanism model of Super-enhancer-associated INSM2 promoting the progression of neuroblastoma (Fig. 7).

## Discussion

Lipids are a highly complex group of biomolecules. It is both an energy source and a structural component of cell membranes, and it can also act as a signaling molecule for energy exchange inside and outside the cell membrane [30]. Abnormal lipid metabolism is one of the most prominent metabolic changes in cancer. In recent years, lipidomics has made many advances in the study of clinical diseases, such as liver cancer [31], breast cancer, prostate cancer [32], colorectal cancer [33]. Lipid metabolism also plays an important role in the early development of many cancers. Enhanced lipid synthesis or uptake contributes to the rapid growth of cancer cells and tumor formation [34].

Activation of fatty acid synthesis occurs in a variety of cancers, and inhibition of fatty acid synthesis shows anti-tumor activity in many tumor types [15], such as ovarian [35], Breast [36], lung [37], colon [38], and other tumors. Neuroblastoma is the second most lethal solid tumor in children, and amplification of the MYCN-oncogene has been shown to be strongly associated with disease progression. However, only a few molecular targets have been identified and used for treatment in the clinic. Studies have shown that neuroblastoma cell viability has decreased significantly after interfering with fatty acid anabolism [25], suggesting that lipid metabolism also plays an important role in neuroblastoma. Inhibition of fatty acid synthesis has been reported to lead to increased neural differentiation and decreased tumor survival. This was also accompanied by a decrease in MYCN or c-MYC oncoprotein levels [25, 39, 40]. Importantly, the prognosis of patients with neuroblastoma is closely related to the expression levels of genes involved in fatty acid synthesis. Therefore, inhibition of adductor fatty acid synthesis is a promising strategy for pharmacological intervention. However, the regulatory networks related to lipid metabolism in neuroblastoma are poorly studied. In our study, INSM2 was a newly identified gene regulating lipid metabolism in neuroblastoma. We first performed in vitro and in vivo experiments by using NB cells knocked down with INSM2. It was verified to play an important role in the proliferation, cycle, and apoptosis of NB cells. Later, the bioinformatics analysis found that its regulated downstream genes were enriched in lipid formation pathways.

Lipid rafts are cholesterol- and sphingolipid-rich microstructural domains located in the cell membrane that are primarily involved in membrane signaling and protein sorting and play an important role in maintaining the biological properties of malignant cells. Sterol regulatory element binding protein1 (SREBP1) is the main transcription factor regulating cholesterol and fatty acid biosynthesis [26]. It binds to SREBP cleavage-activating protein (Scap) and Insig, and it is found in the precursor of the endoplasmic reticulum membrane [41]. If cytosterol levels fall, Insig is separated from the complex and SREBP/Scap is transported to the Golgi apparatus where the precursor is cleaved to release the mature form. The mature form enters the nucleus and transactivates the target gene. In tumor cells, SREBP-1 and − 2 regulate both cholesterol and fatty acid biosynthesis [42]. Sterols are the most common regulator of SREBPs, but recent studies have also shown that SREBPs are also regulated by mTOR signaling [27]. This signal is frequently over-activated in cancer. For example, melanoma antigen ganglioside GD3 is expressed in most melanoma cells [43] and mucin oncoprotein 1 (MUC1) is a glycoprotein that is overexpressed in most breast cancers. They affect cholesterol production and lipid raft integrity in tumor cells through mTOR-induced expression of adipogenic enzymes regulated by SREBPs [44].

We found that INSM2 knockdown was followed by suppression of mTOR phosphorylation levels in neuroblastoma cells and different decreases in important genes involved in the ab initio synthesis of fatty acids (FASN, ACC, ACSS2 and SCD), whose abnormal expression plays an important role in tumor survival. Reducing fatty acid utilization by blocking the fatty acid synthesis pathway has been shown to have antitumor activity in a variety of cancers [45, 46]. We then used fat metabolism inhibitor Fatostatin and DMSO-treated NB cells as positive controls, and verified by flow analysis as well as immunofluorescence that the level of lipid droplets in NB cells decreased significantly after INSM2 knockdown. To further reveal the molecular mechanism of INSM2 inhibition of lipid metabolism in NB cells, we performed quantitative lipidome analysis on NB cells after knockdown of INSM2 and found that molecules involved in lecithin (TAG, DAG, PE, PC), and sphingomyelin (Ceramide, GlcCer) production were significantly decreased. All these suggest that INSM2 plays an important role in the regulation of lipid formation in tumor cells and is a key factor in tumorigenesis. In summary, we suggest that INSM2 affects the expression of SREBP1 by regulating the mTOR signaling pathway, which in turn affects the lipid metabolism process of neuroblastoma cells.

Furthermore, according to the analysis of clinical data, high expression of genes involved in de novo fatty acid synthesis (FASN, ACC, ACSS2 and SCD) was associated with poor prognosis, and INSM2 expression was positively correlated with the expression of these genes. MYCN status inside NB limits current clinical application, but multiple studies have demonstrated that inhibition of fatty acid synthesis induces differentiation of neuroblastoma independent of MYCN status. So, the development of specific inhibitors of fatty acid synthesis may provide an avenue to anti-neuroblastoma. Our findings point to a new upstream target for fatty acid metabolism inhibition in NB. In conclusion, we linked INSM2 with lipid metabolism in NB, and while revealing the mechanism of INSM2-induced NB growth regulation, we also expanded the function of INSM2 in the regulation of NB metabolism, which should have similar mechanisms in other types of tumors and deserves further investigation.

## Materials and methods

### Cell culture

Cell lines were bought from Shanghai, China’s National Collection of Authenticated Cell Cultures and cultivated at 37 °C in a humidified incubator with 5% CO_2_ and examined free of mycoplasma regularly. Short tandem repeat analysis was used to verify all cell lines. Additional file 6 contained information on all cell lines.

### Super-enhancer identification

Enhancers were characterized as H3K27Ac peaks 2 kb distant from any transcriptional start sites using the ROSE (Rank Order of Super Enhancers) method [5]. A cutoff at the inflection point (tangent slope = 1) based on the ranking order after stitching enhancer components together when aggregated within a distance of 12.5 kb was used to identify typical-enhancers and super-enhancers (SEs).

### Luciferase reporter assay

Candidate DNA areas were PCR amplified and cloned into the pGL3-Basic luciferase reporter vector or the pGL3-Promoter firefly luciferase reporter vector (Promega). For normalization, a Renilla luciferase control vector was co-transfected. Lipofectamine 3000 was used to transfect SK-N-B2(2) cells. The enhancer’s luciferase activity was evaluated using the Dual-Luciferase after 72 h of transfection.

### qRT-PCR analysis

The RNeasy Mini Kit was used to produce total RNA. For reverse transcription, Prime Script RT Master Mix was used. Following that, qPCR was performed on the LightCycler 480 Real Time System using the LightCycler 480 SYBR Green I Master mix (Roche). In Additional file 7, the primer sequences are listed.

### Western-blot analysis

The primary antibodies used in this experiment were listed as follows: INSM2 (ab91568, Abcam), MYCN (9405 S, CST), ANXA2 (ab185957, Abcam), FASN (10624-2-AP, Proteintech), ACC (3676 S, CST), ACSS (3658 S, CST), SCD (2794 S, CST), SREBP1 (ab3259, Abcam), mTOR (2983 S, CST), Phospho-mTOR (5536 S, CST) and GAPDH (AP0063, Bioworld Technology Inc.).

### Flow cytometry

NB cells were frozen in 70% cold ethanol and refrigerated overnight at 4 °C after transfection with shRNA for cell cycle determination. At 37 °C for 30 min, the cells were stained with propidium iodide (Sigma) solution. Flow cytometry was used to examine cells (Beckman Gallios). FlowJo Tree Star software was used to evaluate data (TreeStar) [47].

For cell apoptosis assay: Cells were resuspended in 1×binding buffer and stained with the FITC-Annexin V Apoptosis Kit for the cell apoptosis assay. Flow cytometry was used to investigate apoptosis (Beckman Gallios).

For analysing of lipid droplet in NB cells: Harvested cells were washed with cold PBS, stained by HCS LipidTOX™ Green Neutral Lipid Stain, according to the manufacturer’s instructions. Lipid droplet was analyzed by flow cytometry.

### RNA-seq and data analysis

Novogene Bioinformatics Technology Co., Ltd.(Beijing, China) handled RNA isolation, library preparation, transcriptome sequencing, and clean data filtering.

### HCS LipidTOX™ staining

NB cells were fixed with 4% paraformaldehyde for 15 min. After washing with cold PBS, cells were stained with HCS LipidTOX™ green neutral lipid stain (Thermo Fisher, H34475) for 15 min at 37 °C. Cell nuclei were counterstained with DAPI (Invitrogen, Carlsbad, CA, USA). Images were acquired using a fluorescence microscope (Olympus, Lake Success, NY, USA).

### Xenograft assay in vivo

Female mice (4 weeks old) purchased from Lingchang BioTech were used for in vivo tumorigenic assay. For each group of 6 mice, 2 × 10^7^ SK-N-BE(2) cells were injected subcutaneously into the armpit after resuspended in 200 µl PBS. After tumor formation, the injected mice were executed at the indicated time points. The Animal Care Committee of Suzhou College approved all animal studies (approval number: CAM-SU-AP#:JP-2018-1).

### Full quantitative lipomics detection of human NB cells

SK-N-BE(2) cells transfection with shRNA were mixed with the quenched agent in a 1:5 volume ratio. At 4℃, 2500 g centrifuge 5 min; Cells were placed into liquid nitrogen, 10s removed and stored in-80℃.The all cells sample were sent to BIOTREE biotechnology, shanghai for full quantitative lipomics detection.

### Data availability

The GEO database now has the RNA-seq data that supports the current study’s conclusions (https://www.ncbi.nlm.nih.gov/geo, with accession number GSE201173). GEO database (https://www.ncbi.nlm.nih.gov/geo, with accession numbers GSE16476, GSE45547, GSE85047, GSE14340, GSE14880, GSE16237, GSE147635 and GSE13136) contains public datasets. The Cancer Cell Line Encyclopedia (CCLE) database contains information on INSM2 mRNA expression. (https://sites.broadinstitute.org/ccle, search keyword “INSM2”).

### Statistical analysis

Statistical analysis was performed using the GraphPad Prism version 8.0.2(GraphPad Software, Inc., USA). (*p < 0.05, **p < 0.01) was considered statistically significant.

Further details of materials and methods are provided in the Supplementary materials and methods (Additional file 8).

## Supplementary information

**Additional file 1: Fig.S1.** INSM2 expression in GN tissue and NB tissue (GSE147635).**Fig. S2**. SK-N-BE(2) and SK-N-SHcells were infected with sh-NC or sh-INSM2 or sh-INSM2+INSM2. The cells were harvestedfor CCK-8 colorimetric assay. **Fig. S3**. IHCanalysis for CD31. **Fig.S4**. Lipid metabolism-related geneFASN affects the growth of NB. **Fig.S5**. A KEGGpathway analysis of the differentially expressed genes. B-C qPCR and Western blot analysis of the expression for oncogenes of NB (MYCN andANXA2) after knockdown of INSM2. **Fig. S6**. A-D Kaplan-Meier curvesindicating the survival of NB patients with high or low FASN, ACC, ACSS2 andSCD expression.

**Additional file 2: **Differentially expressed genes in INSM2-knockdown SK-N-BE(2) cells with abs(log2(foldchange))>0.5 and adjusted p value<0.05.

**Additional file 3: **KEGG pathway analysis for DEGs in INSM２-knockdown SK-N-BE(2) cells with abs(log2(foldchange))>1 and adjusted p value<0.05.

**Additional file 4: **INSM２, FASN and ACC levels in NB patients.

**Additional file 5**: Full quantitative lipomics detection of SK-N-BE(2) cells.

**Additional file 6**: The information ofall cell lines used in the study.

**Additional file 7: **List of all primers used in the study.

**Additional file 8**: Supplementary materials and methods.

## Data Availability

The datasets used and/or analyzed during the current study are available from the corresponding author on reasonable request. RNA-seq and original data have been submitted to the GEO database with accession number GSE201173.

## Abbreviations

NB: Neuroblastoma
SE: Super-enhancer
CCK-8: Cell Counting Kit- 8
shRNA: Short hairpin RNA
RNA-seq: RNA sequencing
H3K27Ac: Histone 3 lysine 27 acetylation
ROSE: Rank Order of Super Enhancers
IHC: Immunohistochemistry
hNCC: Human neural crest cells
DAG: Diacylglycerol
TAG: Triglyceride
CDP-DAG: Cytidine-diphosphate-diacylglycerol
PE: Phosphatidylethanolamines
PC: Phosphatidylcholine
SM: Sphingomyelinand
GlcCer: Glucosylceramides
GSEA: Gene Set Enrichment Analysis
CCLE: The Cancer Cell Line Encyclopedia
