# Supplementary material for: Super-enhancer-associated INSM2 regulates lipid metabolism by modulating mTOR signaling pathway in neuroblastoma
Source: Cell Biosci. 2022 Sep 16;12:158. doi: 10.1186/s13578-022-00895-3 (PMC9482322; doi:10.1186/s13578-022-00895-3)
Supplement: Supplementary file 1 — Additional file 1: Fig.S1. INSM2 expression in GN tissue and NB tissue (GSE147635).Fig. S2. SK-N-BE(2) and SK-N-SHcells were infected with sh-NC or sh-INSM2 or sh-INSM2+INSM2. The cells were harvestedfor CCK-8 colorimetric assay. Fig. S3. IHCanalysis for CD31. Fig.S4. Lipid metabolism-related geneFASN affects the growth of NB. Fig.S5. A KEGGpathway analysis of the differentially expressed genes. B-C qPCR and Western blot analysis of the expression for oncogenes of NB (MYCN andANXA2) after knockdown of INSM2. Fig. S6. A-D Kaplan-Meier curvesindicating the survival of NB patients with high or low FASN, ACC, ACSS2 andSCD expression. [file 13578_2022_895_MOESM1_ESM.docx]

**Additional File 1：Fig. S1-S5**

**Fig. S1**

**
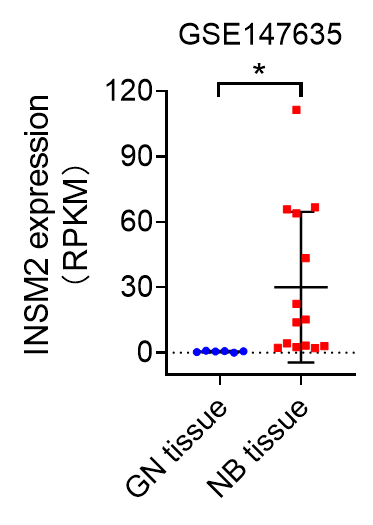
**

**Fig. S1.** INSM2 expression in GN tissue and NB tissue (GSE147635).

**Fig. S2**

**
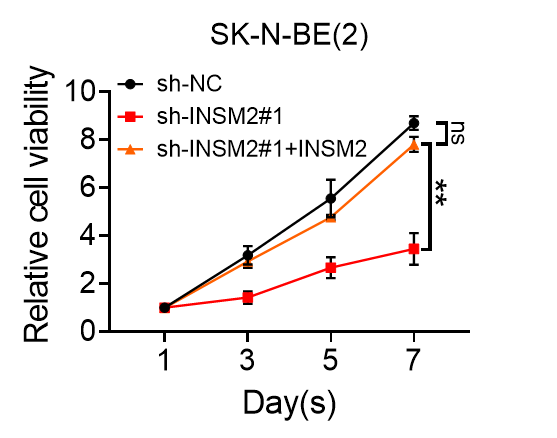

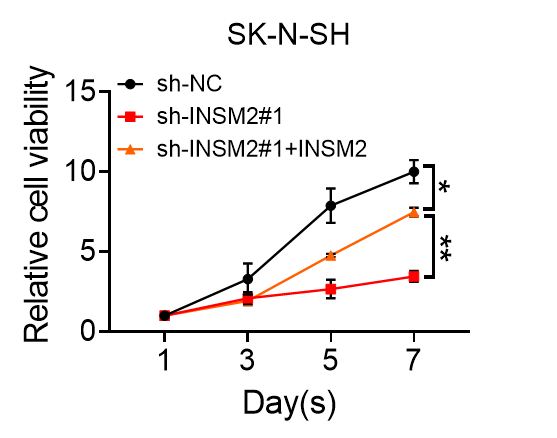
**

**Fig. S2.** SK-N-BE(2) and SK-N-SH cells were infected with sh-NC or sh-INSM2 or sh-INSM2+INSM2. The cells were harvested for CCK-8 colorimetric assay.

**Fig. S3**


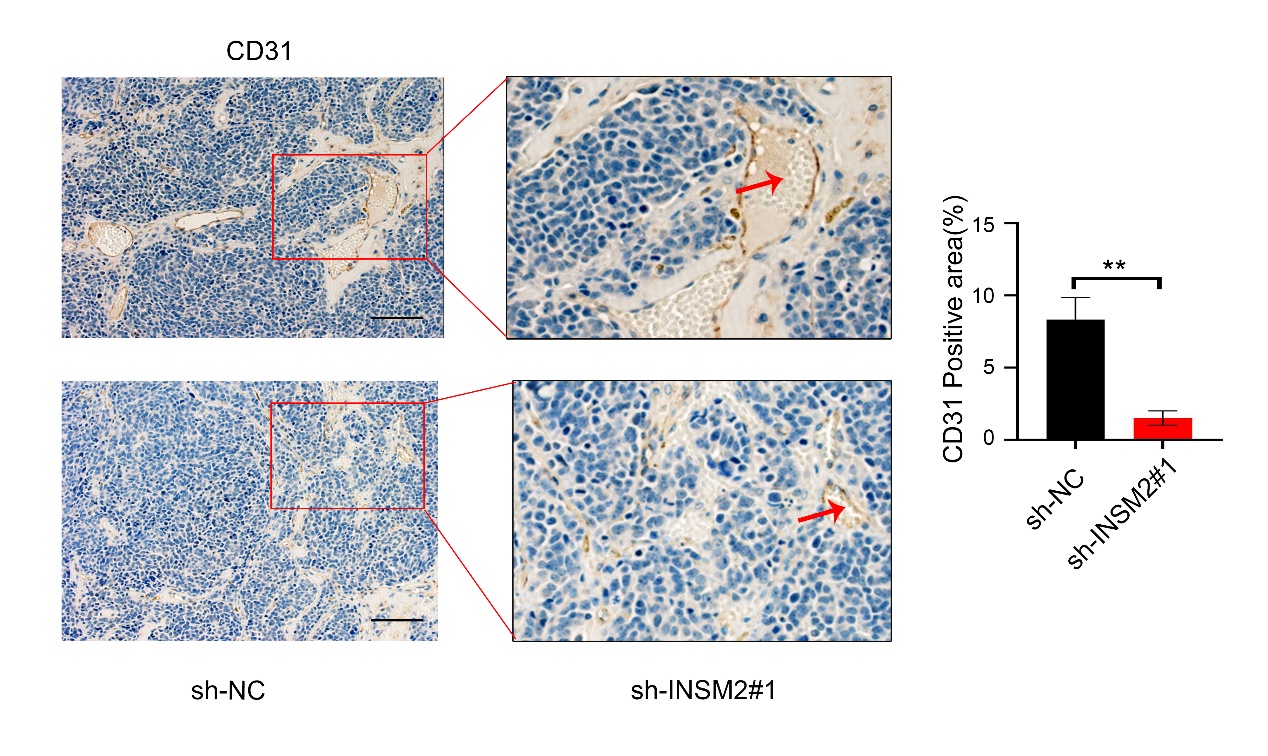
 **Fig. S3.** IHC analysis for CD31.

**Fig. S4**


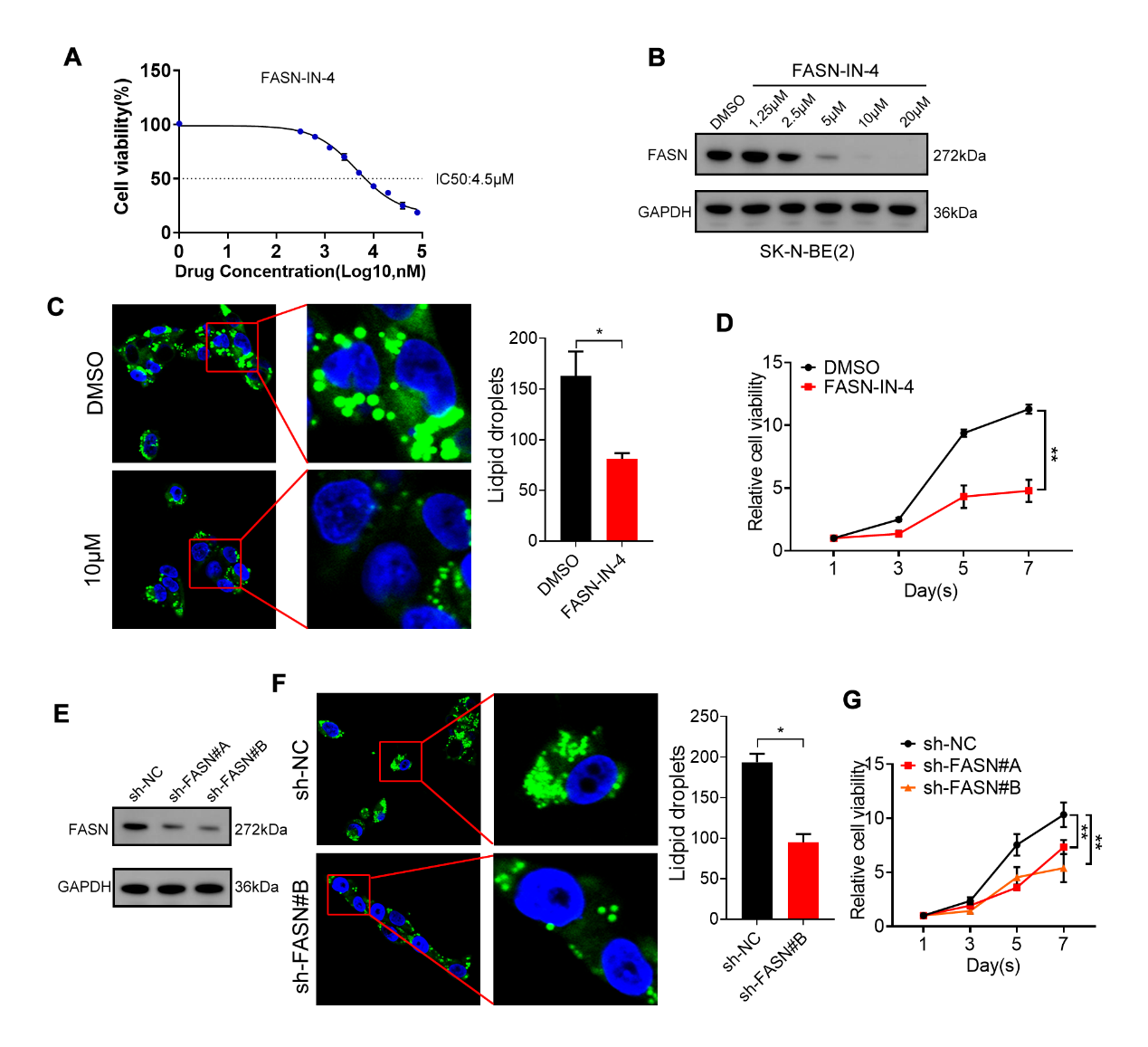


**Fig. S4. Lipid metabolism-related gene FASN affects the growth of NB. A** Cell viability and the IC50 value of NB cells treated with escalating dosages of FASN-IN-4 for 48 hours. **B** Western blotting analysis of FASN levels in NB cells treated with increasing doses of FASN-IN-4 for 48 h. **C** Confocal images(left) of lipid droplet and quantification(right) in SK-N-BE(2) cells treated with FASN-IN-4. **D** SK-N-BE(2) cells were treated with FASN-IN-4 or DMSO. The cells were harvested for CCK-8 colorimetric assay. **E** Western blotting analysis of FASN levels in NB cells infected with sh-NC or sh-FASNs. **F** Confocal images(left) of lipid droplet and quantification (right) in SK-N-BE(2) cells infected with sh-NC or sh-FASNs. **G** SK-N-BE(2) cells were infected with sh-NC or sh-FASNs. The cells were harvested for CCK-8 colorimetric assay.

**Fig. S5**


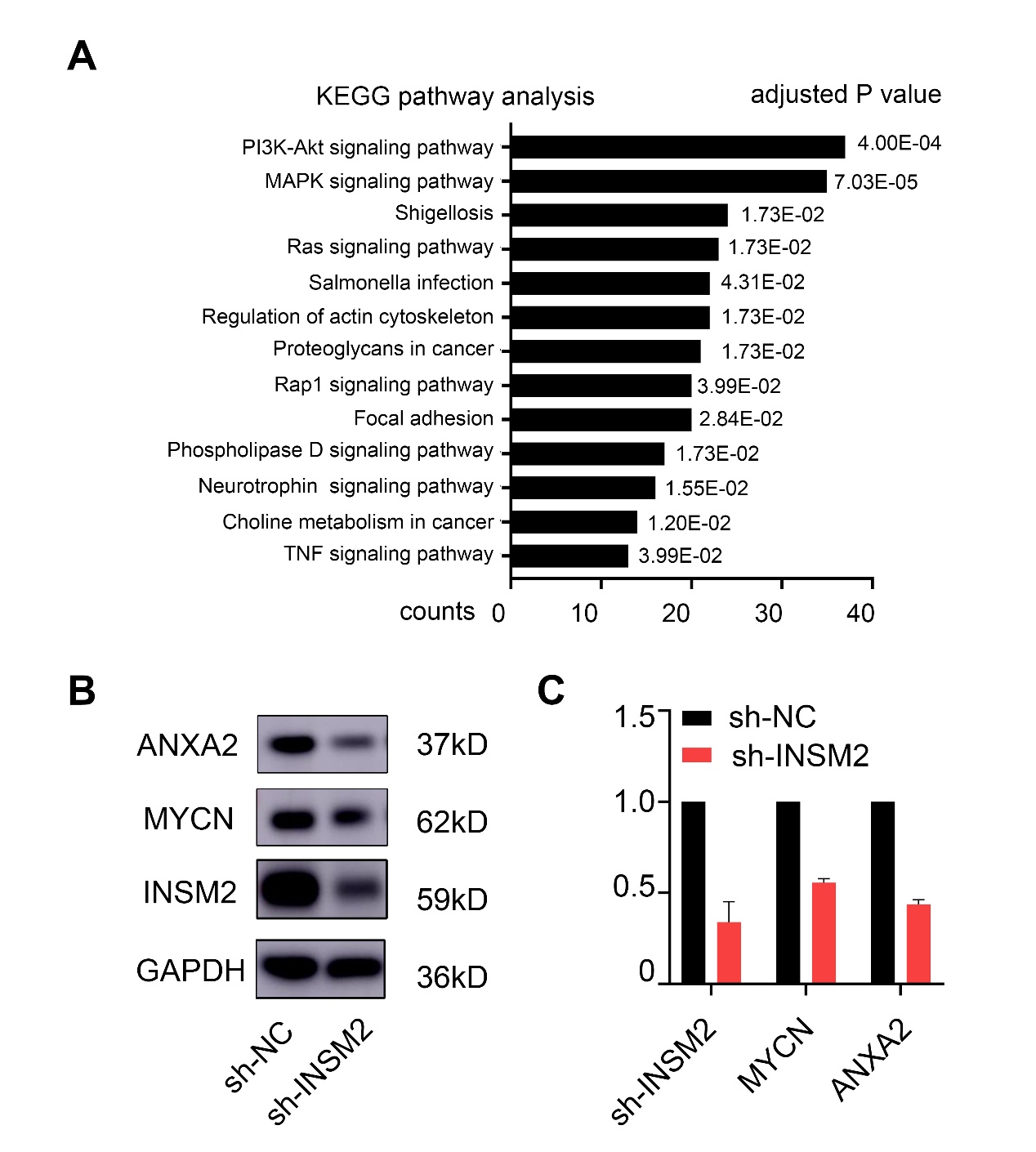


**Fig. S5. A** KEGG pathway analysis of the differentially expressed genes. **B-C** qPCR and Western blot analysis of the expression for oncogenes of NB (MYCN and ANXA2) after knockdown of INSM2.

**Fig. S6**


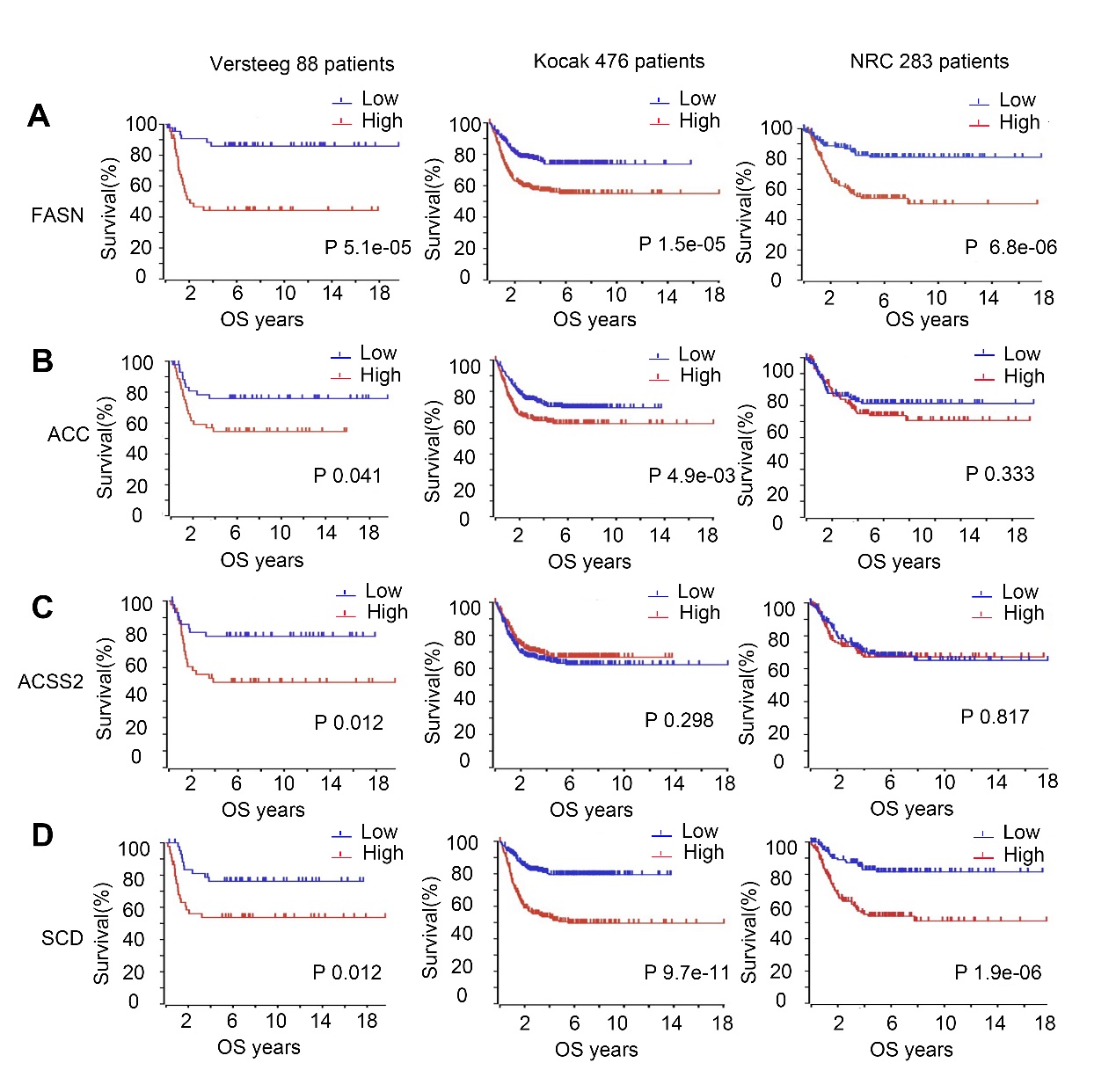
 **Fig. S6. A-D** Kaplan-Meier curves indicating the survival of NB patients with high or low FASN, ACC, ACSS2 and SCD expression.
