# Supplementary material for: Super-enhancer-associated INSM2 regulates lipid metabolism by modulating mTOR signaling pathway in neuroblastoma
Source: Cell Biosci. 2022 Sep 16;12:158. doi: 10.1186/s13578-022-00895-3 (PMC9482322; doi:10.1186/s13578-022-00895-3)
Supplement: Supplementary file 6 — Additional file 6: The information ofall cell lines used in the study. [file 13578_2022_895_MOESM6_ESM.docx]

Additional File 6： The information of all cell lines used in the study.

| **name** | **company** | **source** | **Homo sapiens** | **cultures** |
| --- | --- | --- | --- | --- |
| SK-N-BE(2) | National Collection of Authenticated Cell Cultures | Neuroblastoma | Human | DME/F12+15% FBS |
| SH-SY5Y | National Collection of Authenticated Cell Cultures | Neuroblastoma | Human | MEM+10% FBS |
| IMR32 | National Collection of Authenticated Cell Cultures | Neuroblastoma | Human | MEM+10% FBS |
| SK-N-SH | National Collection of Authenticated Cell Cultures | Neuroblastoma | Human | DMEM+10% FBS |
| 293FT | National Collection of Authenticated Cell Cultures | Kidney | Human | DMEM+10% FBS |
| SBC-2 | National Collection of Authenticated Cell Cultures | [small-cell lung carcinoma](file:///C:/Users/admin/AppData/Local/Programs/baidu-translate-client/resources/app.asar/app.html#/#) | Human | RPMI-1640+10% FBS |
| NCI-H446 | National Collection of Authenticated Cell Cultures | [small-cell lung carcinoma](file:///C:/Users/admin/AppData/Local/Programs/baidu-translate-client/resources/app.asar/app.html#/#) | Human | RPMI-1640+10% FBS |
| NCI-H209 | National Collection of Authenticated Cell Cultures | [small-cell lung carcinoma](file:///C:/Users/admin/AppData/Local/Programs/baidu-translate-client/resources/app.asar/app.html#/#) | Human | RPMI-1640+10% FBS |
| NCI-H69 | National Collection of Authenticated Cell Cultures | [small-cell lung carcinoma](file:///C:/Users/admin/AppData/Local/Programs/baidu-translate-client/resources/app.asar/app.html#/#) | Human | RPMI-1640+10% FBS |
| ESO26 | National Collection of Authenticated Cell Cultures | Esophageal adenocarcinoma | Human | RPMI-1640+10% FBS |
| OACP4C | National Collection of Authenticated Cell Cultures | Gastric cancer | Human | Ham's F-12＋10% FBS |
| AGS | National Collection of Authenticated Cell Cultures | Gastric cancer | Human | Ham's F-12＋10% FBS |
| HGC27 | National Collection of Authenticated Cell Cultures | Gastric cancer | Human | RPMI-1640＋20% FBS |
| MGC803 | National Collection of Authenticated Cell Cultures | Gastric cancer | Human | RPMI-1640＋20% FBS |
| HOS | National Collection of Authenticated Cell Cultures | Osteosarcoma | Human | MEM＋10% FBS |
