# Supplementary material for: Super-enhancer-associated INSM2 regulates lipid metabolism by modulating mTOR signaling pathway in neuroblastoma
Source: Cell Biosci. 2022 Sep 16;12:158. doi: 10.1186/s13578-022-00895-3 (PMC9482322; doi:10.1186/s13578-022-00895-3)
Supplement: Supplementary file 7 — Additional file 7: List of all primers used in the study. [file 13578_2022_895_MOESM7_ESM.docx]

| **Additional File 7： List of all primers used in the study.** | | |
| --- | --- | --- |
| **Gene name** |  | Primers Sequence |
| INSM2 | F | CGAGAATGGCTTGGAGAAAACA |
|  | R | GAAGCTGTCAGCGTTGACCGTG |
| MYCN | F | ACCCGGACGAAGATGACTTCT |
|  | R | CAGCTCGTTCTCAAGCAGCAT |
| ANXA2 | F | GAGCGGGATGCTTTGAACATT |
|  | R | TAGGCGAAGGCAATATCCTGT |
| FASN | F | GCTGGAAGGAGGAAGAGGTT |
|  | R | CTCGAGTGGTCCGTGAGTTT |
| ACC | F | CCCTTGCACATACACAATGC |
|  | R | GGATGGAGGTGGGGTTAAAT |
| ACSS2 | F | AGCGAGCATCCCCCAAAGTT |
|  | R | GGGCACGAAGGCTCATCATT |
| SCD | F | CTCAGTTCCTACGCTTCGCAT |
|  | R | GTCGAGGTCAGTGAACAGCA |
| **Gene name** |  | **shRNA sequences** |
| sh INSM2#1 |  | CCGGGTGTGCCCATATTGCCACAAACTCGAGTTTGTGGCAATATGGGCACACTTTTT |
| sh INSM2#2 |  | CCGGGAAAGGAGAACAGCCGAATAGCTCGAGCTATTCGGCTGTTCTCCTTTCTTTTT |
| sh INSM2#3 |  | CCGGGCCATGAGGAAGTTGAGCTTTCTCGAGAAAGCTCAACTTCCTCATGGCTTTTT |
| sh FASN#A |  | CCGGGCGGCAACGTGGGCATCAACTCTCGAGAGTTGATGCCCACGTTGCCGCTTTTTGAATT |
| sh FASN#B |  | CCGGGGGCCAAACCCAAGCTGATGTCTCGAGACATCAGCTTGGGTTTGGCCCTTTTTGAATT |
