# Supplementary material for: Super-enhancer-associated INSM2 regulates lipid metabolism by modulating mTOR signaling pathway in neuroblastoma
Source: Cell Biosci. 2022 Sep 16;12:158. doi: 10.1186/s13578-022-00895-3 (PMC9482322; doi:10.1186/s13578-022-00895-3)
Supplement: Supplementary file 8 — Additional file 8: Supplementary materials and methods. [file 13578_2022_895_MOESM8_ESM.docx]

**Additional File 8：Supplementary materials and methods**

**Cell proliferation measurement**

48 hours after transfection with siRNA, SK-N-BE(2) and SK-N-SH cells were seeded in 96-well plate (2x10^3^per well). The number of viable cells at different time periods was counted by the Cell Counting Kit-8 (CCK-8) Assay Kit (Beyotime, C0038) according to the manufacture's instructions. In brief, 200uL CCK-8 solution were added into each well and incubated for 1 h at 37℃. The absorbance at 450 nm was read using the MRX Microplate Reader (Dynex Technologies). Experiments were conducted at least 3 times with 3 replicates for each group.

**Colony formation**

Briefly,NB cells were added to the 6-well plates at 2000 cells per well with 2 ml medium and cultured at 37°C for 2 weeks. Cells were fixed in 4% Paraformaldehyde (PFA), and stained with 0.1% Crystal Violet (C8470, Solarbio, Beijing, China). Total number of colonies per well were counted. Three independent replicates were performed.

**Immunohistochemistry (IHC)**

The slides were deparaffinized in xylene, then dehydrated with graded ethanol after 1 hour in an incubator at 60 °C. The antigen was then extracted using a boiling citrate buffer (0.01 M, pH 6.0). After three 5-minute rinses in PBS (pH 7.5), Then, use the UltraSensitive SP (Mouse/Rabbit) IHC Kit and DAB Plus Kit to complete the procedure. Each tissue section's staining results were viewed using an Olympus BX41 imaging equipment, and the proportion of positive cells (P) was multiplied by the intensity of the staining (I). The following antibodies were used: INSM2 (ab91568, Abcam), Ki-67 (GB111499, Servicebio), Cleaved Caspase-3(GB11009-1, Servicebio), CD31(GB13428, Servicebio).

**Full quantitative lipomics detection of human neuroblastoma cells**

were homogenized 60s with 400 μL water. 190μL of water was mixed with the 10μL of homogenate, and then 480μL of internal standard extract was added. The samples were vortexed for 60 s and then sonicated in an ice-water bath for 10 min. centrifuged at 4°C for 15 min at 3000 rpm. 250 μL of supernatant was transferred to a new test tube. The remaining samples were added to 250 μL MTBE, then vortexed, sonicated, centrifuged, and then 250 μL of supernatant was removed. Repeat twice. The supernatant was mixed and dried in a vacuum concentrator at 37°C.The dried samples were reconstituted in 100 μL of resuspension buffer (DCM: MeOH: H2O = 60:30:4.5) and sonicated on ice for 10 min. The supernatant was then centrifuged at 12,000 rpm for 15 min at 4 °C and 30 μL was transferred to a fresh glass vial for LC/MS analysis. Quality control (QC) samples were prepared by mixing equal amounts of supernatant.

The UHPLC separation was carried out using a SCIEX ExionLC series UHPLC System. The mobile phase A consisted of 40% water, 60% acetonitrile, and 10 mmol/L ammonium formate. The mobile phase B consisted of 10% acetonitrile and 90% isopropanol, and 10 mmol/L ammonium formate. The column temperature was 40 ℃. The auto-sampler temperature was 6 ℃, and the injection volume was 2 μL.

AB Sciex QTrap 6500+ mass spectrometer was applied for assay development. Typical ion source parameters were: IonSpray Voltage: +5500/-4500 V, Curtain Gas: 40 psi, Temperature: 350℃, Ion Source Gas 1:50 psi, Ion Source Gas 2: 50 psi, DP: ±80V.

Skyline 20.1 Software was employed for the quantification of the target compounds. The absolute content of individuals lipids corresponding to the IS was calculated on the basis of peaks area and actual concentration of the identical lipid class internal standard (IS), and then absolute content was obtained from diverse internal standard (IS) averaged of the identical lipid class.
